# Modification of Ad5 Hexon Hypervariable Regions Circumvents Pre-Existing Ad5 Neutralizing Antibodies and Induces Protective Immune Responses

**DOI:** 10.1371/journal.pone.0033920

**Published:** 2012-04-05

**Authors:** Joseph T. Bruder, Elena Semenova, Ping Chen, Keith Limbach, Noelle B. Patterson, Maureen E. Stefaniak, Svetlana Konovalova, Charlie Thomas, Melissa Hamilton, C. Richter King, Thomas L. Richie, Denise L. Doolan

**Affiliations:** 1 Research, GenVec, Inc., Gaithersburg, Maryland, United States of America; 2 US Military Malaria Vaccine Program, Naval Medical Research Center, Silver Spring, Maryland, United States of America; 3 Queensland Institute of Medical Research, Brisbane, Australia; French National Centre for Scientific Research, France

## Abstract

The development of an effective malaria vaccine is a high global health priority. Vaccine vectors based on adenovirus type 5 are capable of generating robust and protective T cell and antibody responses in animal models and are currently being evaluated in clinical trials for HIV and malaria. They appear to be more effective in terms of inducing antigen-specific immune responses as compared with non-Ad5 serotype vectors. However, the high prevalence of neutralizing antibodies to Ad5 in the human population, particularly in the developing world, has the potential to limit the effectiveness of Ad5-based vaccines. We have generated novel Ad5-based vectors that precisely replace the hexon hypervariable regions with those derived from Ad43, a subgroup D serotype with low prevalence of neutralizing antibody in humans. We have demonstrated that these hexon-modified adenovectors are not neutralized efficiently by Ad5 neutralizing antibodies *in vitro* using sera from mice, rabbits and human volunteers. We have also generated hexon-modified adenovectors that express a rodent malaria parasite antigen, *Py*CSP, and demonstrated that they are as immunogenic as an unmodified vector. Furthermore, in contrast to the unmodified vector, the hexon-modified adenovectors induced robust T cell responses in mice with high levels of Ad5 neutralizing antibody. We also show that the hexon-modified vector can be combined with unmodified Ad5 vector in prime-boost regimens to induce protective responses in mice. Our data establish that these hexon-modified vectors are highly immunogenic even in the presence of pre-existing anti-adenovirus antibodies. These hexon-modified adenovectors may have advantages in sub-Saharan Africa where there is a high prevalence of Ad5 neutralizing antibody in the population.

## Introduction

Recombinant adenoviruses have been developed as an efficient, replication-defective gene delivery vector [1], [2], [3], [4], [5]. Vaccination with adenovirus type 5 (Ad5) vectors carrying disease-specific antigens results in the induction of broad and protective T cell and antibody responses in a number of animal models, ranging from mouse to non-human primate [6], [7], [8], [9], [10], [11], [12], [13], [14], [15], [16]. In one example, an Ad5 vector encoding the Ebola surface glycoprotein generated neutralizing antibodies and protected monkeys following a single administration [10]. Protection from malaria has also been observed using adenovectors that express the circumsporozoite (CSP) antigen in the *Plasmodium yoelii* [12], [17], [18] or *Plasmodium berghei* [19] murine models. Adenovectors are currently being evaluated in clinical trials for vaccines against HIV [20], [21], [22], tuberculosis [23], and malaria [24], [25], [26]. The Ad5-based HIV and malaria vaccines have been well tolerated and induced antigen-specific CD4+ T cell, CD8+ T cell, and antibody responses in a majority of volunteers [20], [25], [26].

Forty to sixty percent of the United States population have neutralizing antibodies to Ad5 [27], [28] and the frequency is even higher in Africa, where malaria is endemic [29], [30], [31]. In animal models, including mice [32] and non-human primates [33], prior exposure to Ad5 diminished T cell responses to the target antigen following immunization with an Ad5 vector. As expected, due to the type specificity of adenovirus neutralizing antibody (NAb) [34], pre-existing Ad5 NAb were not effective in reducing vaccine-induced responses to other adenovirus vaccines based on alternative human [27], [35] or simian [36], [37], [38], [39] serotypes. Unfortunately, adenovectors derived from alternative human or simian serotypes have generally underperformed Ad5-based vectors with regard to induction of antigen-specific immune responses [3], [17], [32], [35], [40], [41].

Reports addressing the impact of pre-existing Ad5 NAb on adenovector vaccine immunogenicity in humans indicate that volunteers possessing high titers of Ad5 NAb were capable of mounting significant antigen-specific humoral [20] and cellular responses [20], [22]. However, the magnitude and frequency of T cell responses in individuals with pre-existing Ad5 neutralizing antibodies was lower than those observed in Ad5-seronegative volunteers. Overall, these results suggest that adenovectors that are not inhibited by pre-existing adenovirus NAb are likely to have an advantage with respect to vaccine efficacy.

Adenovirus virions are composed of non-enveloped icosahedral shaped capsids that surround the viral DNA-protein core complex. Hexon is the most abundant structural protein, with 240 copies of the trimeric molecule per capsid. Twelve copies of the hexon trimer form each of the 20 triangular facets of the capsid. A complex of penton base and fiber proteins seals the vertices of the capsid and facilitates attachment (fiber) and internalization (penton base) of the virus [42]. Adenovirus-specific NAb have been identified that target hexon, fiber and penton base [43], [44]. However, studies with chimeric adenoviruses have shown that the hexon is the major target for NAb that functions *in vivo* [31], [45], [46]. These findings suggest the potential for generating chimeric capsids composed of hexons from different serotypes as a way of generating adenovectors that avoid pre-existing Ad5 NAb. Ad5 vectors are capable of incorporating hexons from other subgroup C serotypes or Ad12, but hexons from other species (for example, Ad4, Ad7, Ad9, Ad13, Ad17 and Ad35) have not been viable in the context of the Ad5 virion [47]. These results suggest serotype-specific interactions between hexon and other capsid components. An alternative strategy for the generation of Ad5 vectors that avoid pre-existing NAb is, therefore, to modify only the regions of the hexon protein that are the targets of serotype-specific NAb.

The hexon protein is highly conserved among the different adenovirus serotypes with the exception of nine hypervariable regions (HVRs) [48]. Analysis of the crystal structures of Ad2 and Ad5 has shown that these HVRs reside in two distinct loops that form the exposed surface of the hexon protein [48], [49], [50]. HVRs 1–6 lie within the DE1 loop and HVRs 7–9 are located within the FG1 loop. Ad5 vectors carrying substitution of the DE1 and FG1 loops from Ad2, or Ad12 are not neutralized efficiently by Ad5 NAb [46], [51] indicating that these hypervariable loops contain the targets of type-specific neutralizing antibodies. One group of researchers has demonstrated that it is possible to replace each of the 9 HVRs of the Ad5 hexon with those derived from Ad48, a rare adenovirus serotype [52]. Induction of transgene-specific immune responses was unaffected by Ad5-specific NAb in murine or non-human primate vaccine models. Here, we have evaluated the potential of novel Ad5-based vectors where the hexon hypervariable regions are replaced with those derived from alternate serotypes with low prevalence of neutralizing antibody in humans, specifically Ad43 (group D) and Ad34 (group B).

## Results

### Ad5 vectors are tolerant of intersubgroup exchanges in the FG1, but not the DE1 surface exposed loops of hexon

**Figure 1:**
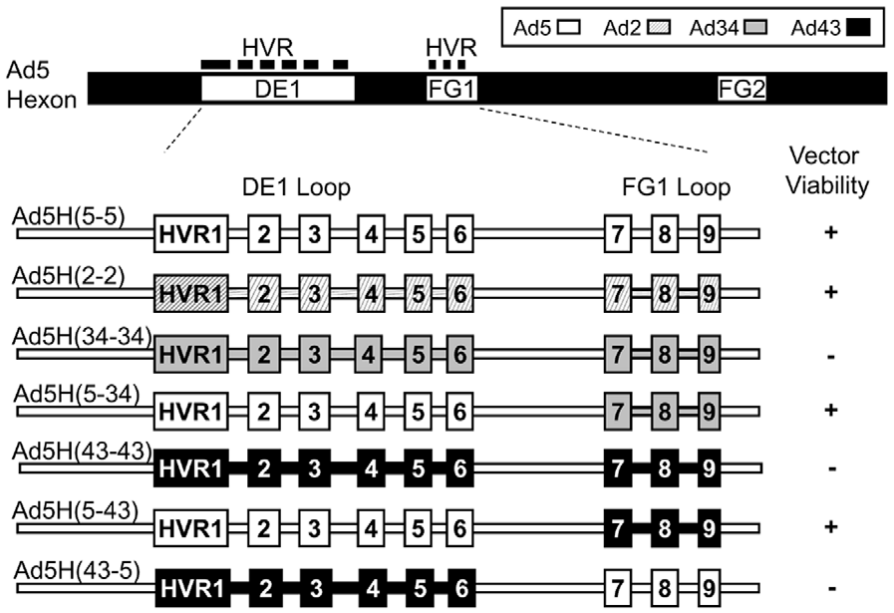
Generation of Ad5 vectors with chimeric hexons derived from Ad2, Ad43 and Ad34. A schematic view of the Ad5 hexon protein and viable chimeric vectors is shown. The positions of surface loops (DE1 and FG1) within the hexon protein are indicated. The HVRs 1–9, indicated in black, are shown above the Ad5 hexon. Sequences from Ad5 are indicated in white, Ad2 in diagonal stripes, Ad34 in gray and Ad43 in black. Viable vectors produced were: Ad5H(5–5) containing the wild-type Ad5 hexon; Ad5H(2–2) containing the Ad2 DE1 and FG1 loops; Ad5H(5–43) containing the FG1 loop from Ad43; and Ad5H(5–34) containing the FG1 loop from Ad34.

We reasoned that substitution of the HVRs in the Ad5 hexon with those derived from less prevalent serotypes would result in a vaccine vector with the potency of Ad5 that would not be inhibited by pre-existing Ad5 NAb. Accordingly we designed and cloned three hybrid vectors with hexon HVRs based on the group C (Ad2); group D (Ad43); and group B (Ad34). The Ad2 HVRs were chosen as a positive control as chimeric hexons derived from Ad2 and Ad5 have been successfully generated previously [46]. Ad34 and Ad43 were chosen due to their low seroprevalence in human populations [27]. Initially, we attempted to substitute the complete Ad5 DE1 loop, containing HVRs 1–6 and the complete FG1 loop containing HVRs 7–9, with comparable loops derived from Ad2, Ad43 and Ad34 (Figure 1 and Figure S1). We successfully rescued recombinant adenovectors carrying the FG1 hypervariable loop substitutions (Figure 1). DE1 loop substitutions were not tolerated to the same degree. Multiple attempts to generate recombinant adenovector from plasmid constructs carrying the Ad43, or Ad34 DE1 loop substitutions did not result in the rescue of viable hybrid vectors. Only the Ad2 DE1 loop exchange was capable of being converted into a virus vector (Figure 1). However, due to the high seroprevalence of Ad2 in human populations [27], generation of chimeric vectors with HVRs from less prevalent adenovector serotypes is preferred.

### Generation of a chimeric Ad5 vector containing all nine hexon HVRs from Ad43

**Figure 2:**
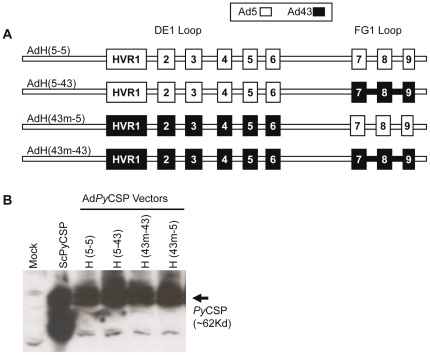
Ad5 vectors containing hexon hypervariable regions from Ad43 express *Py*CSP transgene efficiently. Generation of Ad5 vectors with hypervariable regions of Ad43. A. Schematic showing the DE1 and FG1 loops of the unmodified Ad5 vector and three chimeric vectors containing Ad43 HVR substitutions in the FG1 loop, DE1 loop and both loops as indicated. B. Western blot showing the expression of the *Py*CSP antigen following infection of A549 cells with 200 pu/cell of purified vectors. Mock represents an uninfected cell lysate and serves as a negative control. ScPyCSP represents purified *Py*CSP protein from *Saccharomyces cerevisiae* and serves as a positive control. Hexon designations are indicated above the associated well (H = hexon) and the serotype source of the DE1 and FG1 loops shown in parentheses (DE1–FG1). Immunoblots were probed with antibody specific for *Py*CSP.

The DE1 loop is composed of six HVRs and their intervening sequences, which are more highly conserved across serotypes. The intervening sequences are more highly conserved between the two group C hexons (Ad2 and Ad5) than they are between Ad5 and the group D or group B hexons (Ad43 or Ad34). This is not the case for the HVRs themselves, which are highly divergent between Ad5 and Ad2. We reasoned that the sequences adjacent to the HVRs within the DE1 loop may be important for maintaining a well structured capsid and that the more homologous Ad2 sequences could function in an Ad5 hexon, but the divergent Ad34 and Ad43 HVR flanking sequences could not. We therefore focused on precisely exchanging the presumed targets of type-specific NAb, the six small HVRs within the DE1 loop of Ad5 hexon, with those derived from Ad43. Using this strategy, we were successful at generating Ad5 vectors carrying HVRs 1–9 from Ad43, and the intervening sequences from Ad5 (Figure 2 and Figure S2). We generated hexon-modified vectors that express *Py*CSP and luciferase for use in immunogenicity and *in vitro* neutralization studies, respectively. Chimeric vectors were generated that contained the Ad43 sequence substitutions in the DE1 loop, the FG1 loop, or both loops. All of these hexon-modified vectors had particle:active particle ratios of <50 which are comparable to vectors with unmodified hexons (Table S1). To assess capsid protein composition, we compared the protein pattern of vectors with unmodified and hexon-modified capsids on silver stained gels. This analysis indicated that the protein composition of our hexon-modified vectors is comparable to wild-type Ad5 as well as Ad5 vectors with wild-type hexons (Figure S3). We analyzed transgene expression following infection of A549 cells. Cells were transduced with 200 particle units (pu) per cell of the unmodified or hexon-modified *Py*CSP expressing vectors. *Py*CSP expression was evaluated 24 hours later by immunoblots from infected cell lysates (Figure 2b). The positive control for this western blot (ScPyCSP) is purified *Py*CSP protein from yeast and the extra band is likely a degradation product of the full length protein. Our results show that comparable levels of *Py*CSP are expressed from the unmodified Ad5 vector and from each of the hexon-modified vectors *in vitro*.

### Ad5 hexon-modified vector containing Ad43 substitutions in all nine HVRs is not neutralized efficiently by Ad5 NAb

**Figure 3:**
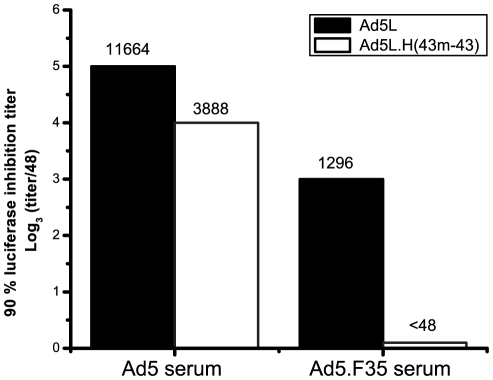
Ad5L.H(43-43) is not neutralized efficiently by Ad5 NAb from mice. Mice were immunized with two administrations (1×10^10^ pu each) of adenovector at an interval of one month. The serum from these mice obtained three weeks after the last immunization was pooled, diluted 1∶48 and then 3-fold serial dilutions were tested for neutralizing activity toward an unmodified Ad5L vector and the Ad5L.H(43 m-43) hexon-modified vector. Two different adenovectors were used to immunize mice and generate serum, an Ad5 vector (Ad5) and an Ad5 vector with an Ad35 fiber replacement (Ad5.F35).

To assess the ability of Ad5 NAb to neutralize the chimeric vector, we generated NAb in mice. Mice were immunized with two administrations (1×10^10^ pu each) of Ad5 vector at an interval of one month and the serum from these mice was tested for neutralizing activity against an unmodified Ad5 vector that expresses luciferase (Ad5L) or the chimeric hexon-modified luciferase vector (Ad5L.H(43 m-43*)*. This serum efficiently neutralized the Ad5 vector but was less effective at neutralizing the hexon-modified vector (Figure 3, Ad5 serum). To determine if the residual neutralizing activity observed in the hexon-modified vector was due to NAb specific for the Ad5 fiber, we generated NAb from mice using an Ad5 vector containing an Ad35 fiber. Serum from these mice neutralized the Ad5 vector, but did not neutralize the hexon-modified vector (Figure 3, Ad5.F(35*)* serum). The neutralizing activity of the full serial dilution series is shown in Figure S4. In control experiments, we immunized mice with Ad43 and observed that these sera neutralized the Ad5L.H(43 m-43), but not Ad5L (data not shown). These data indicate that neutralizing antibodies specific for both the hexon HVRs and the fiber are functional in *vitro*.

**Figure 4:**
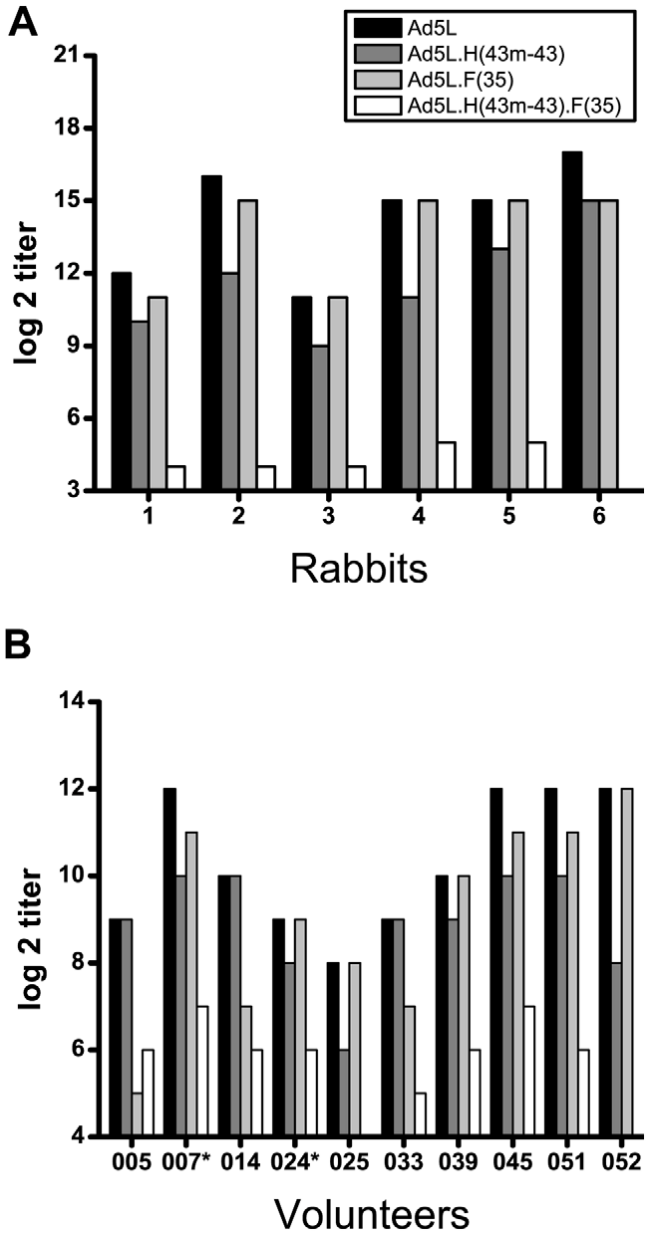
NAb directed at both HVRs and fiber are dominant *in vitro*. Sera from (A) rabbits immunized with two administrations of 1×10^10^ pu of AdNull or (B) human volunteers screened for the presence of neutralizing antibodies specific for Ad5 were analyzed for NAb titers against a panel of chimeric adenovectors expressing the luciferase reporter gene. Ad5L is an unmodified Ad5 vector; Ad5L.H(43 m-43) is a hexon-modified Ad5 vector containing HVRs 1–9 from Ad43; Ad5L.F35 is an Ad5 vector carrying the Ad35 fiber; Ad5L.H(43 m-43).F35 is a hexon-modified vector carrying the Ad35 fiber. The asterisk indicates serum samples that scored positive for Ad35-specific NAb.

To expand the *in vitro* vector neutralization studies, we analyzed NAb responses from rabbits and humans against a panel of chimeric adenovectors (Figure 4). Four luciferase expressing Ad5 vectors were analyzed to differentiate NAb responses specific for the Ad5 HVRs, the Ad5 fiber, or other Ad5 capsid components. Ad5L has a wild-type Ad5 capsid. Ad5L.H(43 m-43) contains nine HVRs from Ad43. Ad5L.F(35) contains the Ad35 fiber. Ad5L.H(43 m-43)F(35) contains nine HVRs from Ad43 and the fiber from Ad35. Rabbits were immunized with two doses (1×10^10^ pu each) of Ad5Null vector at a one month interval and the serum from individual rabbits was harvested one month after the last administration and tested for its capacity to neutralize the chimeric adenovectors. Neutralization titers for Ad5L ranged from 2,048 to 131,072. In all six rabbits, Ad5L.H(43 m-43) was neutralized less efficiently than Ad5L (p<0.05) indicating the existence of NAb specific for the Ad5 HVRs. There was a trend toward reduced neutralization of Ad5L.F(35) relative to Ad5L. In 50% of the rabbits (rabbits 1, 2 and 6), Ad5L.F(35) was neutralized less efficiently than Ad5L indicating the existence of NAb specific for the Ad5 fiber in these animals (Figure 4a). However, in the other 50% of the animals (rabbits 3, 4 and 5), Ad5L.F(35) and Ad5L were neutralized equally well indicating that all of the neutralization measured in this assay can be accounted for by non-fiber capsid components. In all six rabbits, there was a dramatic drop in NAb titer to Ad5L.H(43 m-43).F(35) relative to Ad5L, Ad5L.H(43 m-43), and Ad5L.F(35) (p<0.05). In one of the six rabbits (rabbit 6), no Ad5L.H(43 m-43).F(35)-specific Nab were detected. These data show that most of the adenovirus NAb generated in rabbits immunized with Ad5 vectors is specific for the hexon HVRs and the fiber and that there is a relatively low concentration of NAb directed to other capsid components. We hypothesize that fiber-specific NAb were not revealed in rabbits 3, 4, and 5 by comparing Ad5L to Ad5L.F(35) because in these animals the titer of the fiber-specific NAb is much lower than that of the hexon-specific NAb. Comparison of the NAb titers for Ad5L.H(43 m-43) and Ad5L.H(43 m-43)F(35), reveals that fiber-specific NAb account for an appreciable component of the total NAb titer when the dominant HVR-specific NAb are rendered non-functional.

Next, we analyzed serum from a population of 25 human volunteers from Gaithersburg, MD (Figure 4b). Ad5-specific NAb were detected in 11 individuals. Serum samples from 10 individuals had titers ranging from 256 to 4096 and one sample had a titer between 16 and 48. Sera from individuals that were negative for Ad5 NAb (titer<16) and from this individual with low titer Ad5 NAb were not included in the analysis shown in Figure 4b. Ad35-specific NAb were detected in four of the 25 individuals, two of which were also positive for Ad5 NAb. Analysis of the capacity of serum NAb to neutralize the chimeric vectors indicated that most of the NAb in humans are directed to the hexon HVRs and fiber and only a minority to other capsid components, consistent with the results in rabbits. Specifically, in most of the volunteers (7/10), Ad5L.H(43 m-43) was neutralized less efficiently than Ad5L (p<0.05) indicating the existence of NAb specific for the Ad5 HVRs. In 6/10 of the volunteers, Ad5L.F(35) was neutralized less efficiently than Ad5L (p<0.05) indicating the existence of NAb specific for the Ad5 fiber in these individuals (Figure 4b). However, in the other volunteers (4/10), Ad5L.F(35) and Ad5L were neutralized equally well indicating that all of the neutralization measured in this assay can be accounted for by non-fiber capsid components. In all 10 volunteers, there was a large drop in NAb titer to Ad5L.H(43 m-43).F(35) relative to Ad5L (p<0.05) indicating that NAb specific for both hexon and fiber dominate the anti-Ad5 NAb response in these volunteers. In some volunteers (039, 052, 024 and 025) fiber-specific NAb are only revealed by rendering hexon-specific NAb non-functional and comparing Ad5L.H(43 m-43).F(35) and Ad5L.H(43 m-43). Similarly, in other volunteers (033, 005, 014) hexon-specific NAb are only revealed by rendering fiber-specific NAb non-functional and comparing Ad5L.H(43 m-43).F(35) and Ad5L.F(35).

### Ad5 hexon-modified vector containing Ad43 substitutions in all nine HVRs induces robust *Py*CSP-specific CD8^+^ T cell responses in mice with high titers of Ad5 specific NAb

**Figure 5:**
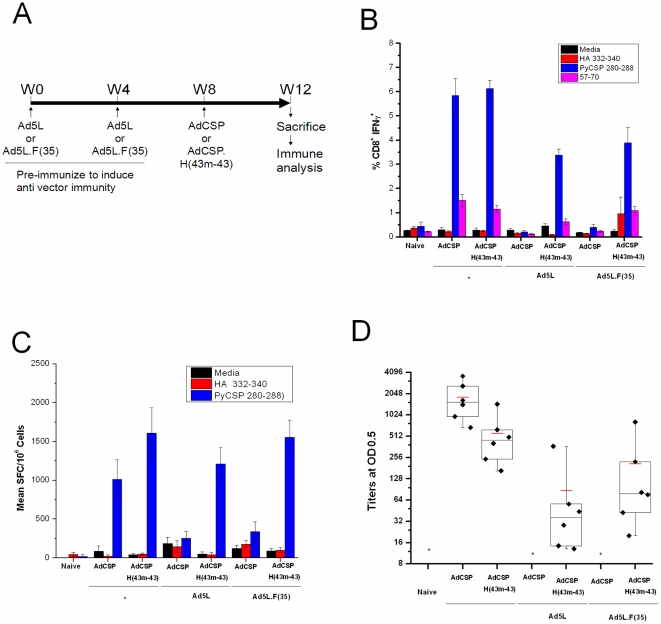
Ad5*Py*CSP.H(43 m-43) induces T cells responses similar to Ad5*Py*CSP in naïve mice and is not inhibited by Ad5 NAb *in vivo*. Naïve BALB/c mice or mice pre-immunized with two injections (1×10^10^ pu each) of Ad5 vector or an Ad5 vector containing the Ad35 fiber, were immunized with Ad5*Py*CSP, or hexon-modified vector Ad5PyCSP.H(43 m-43) (n = 6 mice/group). *Py*CSP-specific T cell responses were assessed by Intracellular cytokine staining (ICS) and IFNγ ELISpot four weeks after immunization. Targets were MHC-matched A20.2J cells pulsed with synthetic peptides representing the immunodominant CD8^+^ T cell epitope (PyCSP280–288) or CD4^+^ T cell epitope with nested subdominant CD8^+^ T cell epitope (*Py*CSP57–70) from *Py*CSP or a defined CD8^+^ T cell epitope for the influenza virus hemagglutinin antigen (HA 332–340). (A) Schematic of the experiment. (B) ICS analysis of CD8^+^ IFNγ^+^ T cell responses from immunized mice assayed individually. Error bars indicate the standard error of the mean, n = 6. (C) IFNγ ELISpot responses from pooled splenocytes (250,000 cells/well). Error bars indicate the standard deviation of the mean from quadruplicate samples. (D) ELISA against recombinant *Py*CSP protein capture antigen. Box-and-whisker plot with values for individual mice shown (n = 6). The mean is indicated by the red line. * indicates groups where titers from all mice were <8.

To assess the immunogenicity of hexon-modified Ad5 vectors expressing *Py*CSP, we compared induction of T cell responses in mice immunized with unmodified and hexon-modified vectors. BALB/c mice were immunized with 1×10^8^ pu of adenovector expressing *Py*CSP (Figure 5a). Antigen specific T cell responses were evaluated by intracellular cytokine staining (ICS) (Figure 5b) and by IFNγ ELISpot (Figure 5c) and *Py*CSP-specific antibody responses were assessed by ELISA (Figure 5d). For both the unmodified and hexon-modified vectors we observed robust induction of *Py*CSP-specific IFNγ^+^CD8^+^ T cell and antibody responses four weeks following immunization. T cell responses were specific for both the immunodominant (*Py*CSP 280–288) and subdominant (*Py*CSP 57–70) CD8^+^ T cell epitopes and there were no statistically significant differences in CSP-specific T cell responses between the unmodified and hexon-modified vectors.

To determine the effect of pre-existing Ad5 NAb on the immunogenicity of these vectors, mice were pre-immunized with two administrations (1×10^10^ pu each), one month apart, of Ad5 vector (Ad5L) or an Ad5 vector containing an Ad35 fiber (Ad5L.F(35)). Under these conditions, the unmodified vector, Ad5*Py*CSP, was substantially reduced in its capacity to induce *Py*CSP-specific T cell and antibody responses as indicated by ICS, ELISpot and ELISA. In contrast, the Ad43 hexon-modified vector, AdCSP.H(43 m-43), induced significantly higher *Py*CSP-specific T cell responses in mice pre-immunized with Ad5 vectors versus the unmodified vector, AdCSP(Figure 5b and c; p<0.05). However, we observed a small but significant reduction in CSP-specific CD8^+^ T cells induced by AdCSP.H(43 m-43) in mice pre-immunized with Ad5 vector relative to naïve mice (p<0.05). The hexon-modified vector also induced *Py*CSP-specific antibody responses in mice pre-immunized with Ad5 vectors, although these responses were reduced relative to the antibody responses induced in mice not previously exposed to Ad5 vectors (Figure 5d). There was no difference in the *Py*CSP-specific T cell or antibody responses induced by *Py*CSP.H(43 m-43) in mice pre-immunized with Ad5 vector or Ad5 vector with the Ad35 fiber, suggesting that antibody to fiber does not reduce the immunogenicity of the Ad5*Py*CSP.H(43 m-43) vector in mice (Figure 5).

### Analysis of immunogenicity and protection from *P. yoelii* sporozoite challenge following immunization of mice with Ad5*Py*CSP.H(43 m-43) and Ad5*Py*CSP in prime-boost regimens

**Figure 6:**
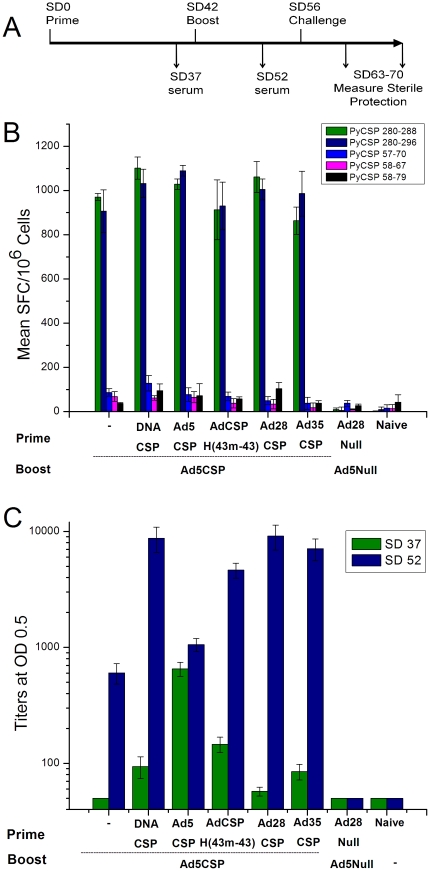
Prime boost immunogenicity studies. BALB/c mice (n = 20 mice/group) were immunized with DNA or adenovectors as indicated. (A) Schematic of the experiment. (B) *Py*CSP-specific T cell responses were assessed from a subset of the mice (n = 6) by IFNγ ELISpot two weeks after immunization. Targets were MHC-matched A20.2J cells pulsed with synthetic peptides representing the immunodominant CD8^+^ T cell epitope (*Py*CSP280–288), CD4^+^ T cell epitope with nested CD8+ T cell epitope (*Py*CSP280–296), the immunodominant CD4^+^ T cell epitope (*Py*CSP57–70), the subdominant CD8^+^ T cell epitope (*Py*CSP58–67), and CD4^+^ T cell epitope with nested CD8+ T cell epitope (*Py*CSP58–79) from *Py*CSP. Negative control values were subtracted from reported data. Error bars indicate the standard deviation of the mean from quadruplicate samples. (C) ELISA against *Py*CSP repeat peptide capture antigen. Error bars indicate the standard error of the mean.

**Figure 7:**
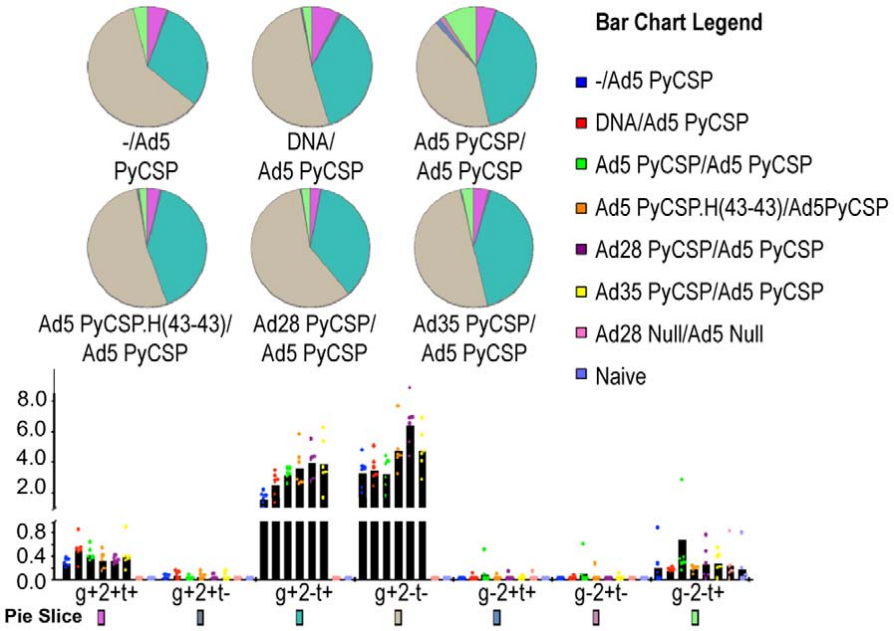
Polyfunctional T cell responses following different vaccine regimens. Splenocytes from mice immunized as in Figure 6 (n = 6) were analyzed for polyfunctional CD8^+^ T cell responses following stimulation with A20.2J cells pulsed with a synthetic peptide representing the immunodominant CD8^+^ T cell epitope (*Py*CSP280–288). Responses were measured by ICS for IFNγ, IL-2 and TNFα. Black bars indicate the mean percentage of cells positive for each combination of cytokines. Responses from individual animals are shown by the colored dots. (g = IFNγ, 2 = IL2, t = TNFα). Pie charts indicate the fraction of the total response comprising cells expressing each of the seven possible combinations of IFNγ, IL-2 and TNFα.

We have demonstrated that a hexon-modified Ad5 vector can effectively boost an unmodified Ad5 vector primed immune response, generating more robust T cell and antibody responses than either vector administered alone when administered at low doses (Figure S5). To determine if an Ad5 hexon-modified prime-Ad5 unmodified boost regimen is capable of inducing protective immune responses, we immunized mice with 1×10^9^ pu of Ad5*Py*CSP.H(43 m-43) followed by a boost six weeks later with Ad5*Py*CSP and measured sterile protection following *P. yoelii* sporozoite challenge (Table 1) as well as T cell and antibody responses (Figures 6 and 7). In parallel, we evaluated mice primed with *Py*CSP expressing plasmid DNA (DNA-*Py*CSP), Ad5*Py*CSP, Ad28*Py*CSP or Ad35*Py*CSP and then boosted with Ad5*Py*CSP. Two weeks after the Ad5*Py*CSP boost, mice were challenged by intravenous (IV) injection with 100 freshly dissected *P. yoelii* sporozoites. Blood stage parasitemia was scored following daily blood smears from day 7–14. Our results show negligible protection with either one or two doses of the Ad5*Py*CSP vector (0% and 14%, respectively). However, we saw statistically significant protection with each of the heterologous prime-boost regimens tested (Table 1). The DNA prime-Ad5 boost regimen protected 36% of mice. There was a trend toward higher levels of protection in the heterologous adenovector prime-boost regimens, with the Ad5*Py*CSP.H(43 m-43) prime-Ad5*Py*CSP boost, Ad28 prime-Ad5 boost and Ad35 prime-Ad5 boost, protecting 43, 54 and 46% of mice, respectively. These data establish that heterologous adenovector prime-boost regimens can induce protective responses in mice and also show that hexon-modified Ad5 vector can be combined with an unmodified Ad5 vector in an effective prime-boost regimen.

**Table 1:** Protective efficacy of adenovector prime-boost regimens.

Prime	Boost	# Protected	# Challenged	% Protected	P value
None	Ad5CSP	0	14	0%	n/a
Ad5CSP	Ad5CSP	2	14	14%	0.1057
DNA-CSP	Ad5CSP	10	28	36%	0.0007
Ad5CSP.H(43 m-43)	Ad5CSP	6	14	43%	0.0006
Ad28CSP	Ad5CSP	15	28	54%	<0.0001
Ad35CSP	Ad5CSP	13	28	46%	<0.0001
Ad28Null	Ad5Null	0	28	0%	1

DNA dose = 100 µg.

Ad dose = 1×10^9^ pu.

Fischer's Exact Test, two-tailed versus Ad Null group.

All vaccine prime-boost regimens tested (DNA/Ad and Ad/Ad) induced robust IFNγ ELISpot responses directed against peptides containing the CD8^+^ dominant epitope (*Py*CSP280–288 and *Py*CSP280–296) (Figure 6b). The heterologous adenovector prime/boost regimens induced *Py*CSP antibody titers comparable to DNA/Ad5-*Py*CSP and higher titers than Ad5/Ad5-*Py*CSP and Ad5-*Py*CSP alone regimens (Figure 6c). Protective regimens (p<0.05) induced higher *Py*CSP-specific antibody titers (>4500 titer at OD 0.5) 10 days post-boost as compared with the non-protective regimens. DNA/Ad5, Ad5.H43/Ad5, Ad28/Ad5, and Ad35/Ad5 titers were comparable and all three superior to Ad5/Ad5 and A5 alone (DNA/Ad5∼Ad28/Ad5∼Ad35/Ad5∼Ad5H43/Ad5>Ad5/Ad5∼−/Ad5, p<0.05). Excepting Ad5/Ad5, *Py*CSP-specific titers were enhanced >30 fold by the boost in the prime-boost regimens (Figure 6c).

ICS analysis confirmed that *Py*CSP-specific CD3^+^CD8^+^ cells were the primary responders, with minimal *Py*CSP-specific CD3^+^CD4^+^ responses (data not shown). Similar to the ELISpot data, the CD8^+^ recall responses to the *Py*CSP280–288 (Figure 7) and *Py*CSP280–296 (data not shown) peptides were robust, but low to the CD4^+^ dominant and CD4^+^ and CD8^+^ subdominant epitopes (data not shown). CD8^+^ responses were largely IFNγ^+^ or IFNγ^+^TNFα^+^ populations. Minor CD8^+^IFNγ^+^TNFα^+^IL2^+^ multifunctional populations were observed (Figure 7), but did not correlate with protection. *Py*CSP-specific CD4^+^ cytokine responses were poor, limited to IFNγ^+^ or TNFα^+^ producers, present in only some mice/group, and were not multifunctional (IFNγ^+^TNFα^+^IL2^+^)(data not shown).

## Discussion

Ad5-based vectors are capable of generating robust and protective T cell and antibody responses in animal models and are currently being evaluated in clinical trials for HIV [20] and malaria [24], [25], [26]. However, the high prevalence of NAb to Ad5 in the human population, particularly in the developing world [29], [30], [31] has the potential to limit the effectiveness of Ad5-based vaccines. The majority of serotype-specific NAb recognize determinants on the hexon and fiber capsid proteins [43], [44], and hexon-specific NAb appear to be the most prevalent and potent *in vivo* [31], [45], [46]. Epitopes targeted by these hexon-specific NAb have been mapped to the hypervariable domains of the hexon protein, contained within exposed loops at the surface of the capsid [46], [53].

We have developed a hexon-modified adenovector vaccine platform for malaria vaccine development that avoids the problem of pre-existing anti-adenovirus immunity prevalent in human populations. We were able to modify the determinants of NAb on the Ad5 hexon so that anti-Ad5 NAb could not neutralize the hexon-modified vector efficiently *in vivo*. Accordingly, we deleted the hypervariable loops from the hexon protein within an Ad5 adenovector vaccine and replaced them with the loops derived from selected serotypes with low prevalence in humans. We substituted the Ad5 FG1 hypervariable loop with loops from Ad2 (group C), Ad34 (group B) and Ad43 (group D) adenoviruses. We also attempted to substitute the large DE1 loop in its entirety with loops from Ad2, Ad34 and Ad43, but only the Ad2 hypervariable loop swap was capable of being converted into a virus vector. Thus, the Ad5 capsid displayed considerable tolerance to modifications in the FG1 loop and is less flexible in accommodating modifications in the DE1 loop. We were able to specifically substitute the HVRs in the Ad5 DE1 loop with Ad43 HVRs by retaining the Ad5 derived non-HVR residues. Thus, we have generated a chimeric Ad5 vector containing all nine HVRs from the non-prevalent Ad43 serotype. Although Ad5 fiber-specific neutralizing antibodies were capable of neutralizing this vector *in vitro*, they did not limit the induction of antigen-specific antibody or T cell responses in mice immunized with this vector. This is consistent with previous reports suggesting that Ad5 fiber-specific neutralizing antibody does not inhibit Ad5 vector induced immune responses [31], [35], [45], [46], [52]. The differential capacity of anti-hexon and anti-fiber NAb to blunt the immunogenicity of Ad5-based vectors *in vivo* is not understood, but may be related to their different mechanisms of action. Anti-hexon NAb function by a rapid single-hit kinetic mechanism and can neutralize virus already attached to cells. In contrast, anti-fiber NAb function with slower kinetics and exclusively function prior to cell attachment [54].

Prime-boost regimens have been used with great success in many disease models and this strategy may be needed to achieve optimal immune responses to prevent or control *Plasmodium* infection. We have previously shown that a booster administration of Ad5 vector was capable of boosting Ad5 primed antigen-specific antibody responses in mice, but did not boost T cell responses [55]. However, Ad5 vectors can efficiently boost T cell responses primed by heterologous Ad vectors, including Ad35 [17]. Here we show that our hexon-modified vector could be paired with an unmodified Ad5 vector in prime-boost regimens and induced more robust T cell responses than either vector by itself when the vectors were administered at low doses. At higher doses of vector (1×10^9^ pu) this heterologous prime-boost regimen provided significant protection in the *P. yoelii* challenge model where the single Ad5 vector or a homologous Ad5 prime-boost regimen did not protect efficiently. However, at these high doses of vector, the heterologous prime-boost regimens did not effectively boost T cell responses relative to a single administration of Ad5.

Previously, Tsuji and colleagues demonstrated that Ad5 vectors expressing *Py*CSP can provide protection against experimental challenge with *P. yoelii* sporozoites [12], [17], [18]. Protection was dependent on CD8+ T cells [12], [17], [18], but not on IFNγ [12], [17], [18] as depletion of CD8+ T cells abrogated protection and the vaccine protected mice deficient in IFNγ or the receptor for IFNγ. Lower doses of Ad5 vectors do not sterilely protect mice from *P. yoelii* challenge. With these low dose immunizations, prime-boost regimens are required. About 50% of the mice in our studies were sterilely protected from sporozoite challenge using the heterologous adenovector prime-boost regimens, which is similar to the level of protection we observed using DNA prime-Ad5 boost regimens. It appears that IFNγ expressing CD8^+^ T cells are not a marker for protection as non-protective regimens including Ad5 vector alone or the homologous Ad5 vector prime-boost regimen induced a similar frequency of IFNγ expressing CD8^+^ T cells as the protective heterologous prime-boost regimens. We also examined expression of TNFα and IL-2 by *Py*CSP antigen-specific T cells and observed no dramatic differences between protected and unprotected groups.

It is clear that antibodies directed against the repeat region of CSP can provide protection against malaria. In rodent and non-human primate models, passive transfer of antibodies directed against the repeat region of *P. berghei*, *P. yoelii*, and *P. vivax* CSPs can protect from experimental challenge [56], [57], [58], [59]. In this study, CSP-specific antibody responses appeared to correlate with protection as regimens that induced the highest levels of protection (heterologous Ad and DNA prime-Ad boost) were associated with higher CSP-specific antibody responses relative to regimens showing poor protective responses (Ad5 vector alone or the homologous Ad5 vector prime-boost regimen). Additional studies need to be performed to determine the correlates of protection following vaccination with these heterologous prime-boost regimens in the *P. yoelii* model.

Our data suggest that hexon-modified vectors may be capable of inducing more robust T cell and antibody responses in people with high titers of Ad5 neutralizing antibody. Data from recent HIV clinical trials indicate that adenovectors that are not inhibited by high titers of pre-existing NAb may have an advantage with respect to induction of T cell responses and vaccine efficacy. Pre-existing Ad5 neutralizing antibody has been shown to reduce the percentage of responders as well as the magnitude of T cell responses in people vaccinated with Ad5-based vaccines [20], [21], [22]. As data in animal models indicate that T cells are required for protection against pre-erythrocytic stage malaria [60], [61], hexon-modified vectors may be more immunogenic and efficacious in people with high titers of Ad5 neutralizing antibody. Because of the high prevalence of NAb to Ad5 in human populations, many groups are pursuing rare human [27], [62] or chimpanzee adenovirus serotypes [36], [37] for vaccine delivery. However, most if not all of the vectors derived from these alternative serotypes have been shown to be less potent than Ad5 in inducing transgene-specific T cell and antibody responses. Hexon-modified Ad5 vectors are a powerful alternative that can provide the potency of Ad5 without the susceptibility to being neutralized by highly prevalent Ad5 NAb. These vectors should be advanced and tested for vaccine efficacy in the human experimental challenge model.

## Materials and Methods

### Advector construction

Adenovirus vectors were generated by using our plasmid-based adenovector construction system. Briefly, the recombinant Ad5 genomes (containing a transgene expression cassette and hexon modification) were liberated from plasmids by digestion with Pac I restriction endonuclease, which cleaves adjacent to the inverted terminal repeats (ITR) of the Ad5 genome. This vector DNA was transfected into monolayer 293-ORF6 cells [1], [2], [3], [4], [5] and cell lysates were serially passaged every three days until cytopathic effect (CPE) was observed. CPE is an early indication that the viral vector is growing in the complementing cell line. The CPE lysates were expanded in four 10 cm plates and the cell lysates harvested three days later. The expanded cell lysates were analyzed to determine infectious vector particle concentration, analyzed by PCR to insure vector genomic integrity and used to seed a production run in suspension 293-ORF6 cells in shaker flasks using serum-free media. Three days after infection the recombinant vectors were released from infected cells by three cycles of freeze-thawing, treated with benzonase, purified by banding on three successive CsCl gradients, dialyzed into final formulation buffer and stored at −80°C. Physical particle units (pu) were determined by absorbance at 260 nm following disruption of the capsid with SDS.

The specific modifications to the Ad5 hexon in the Ad5H(43 m-43) vectors is described below. The HVR1 residues from Ad5 between and including AA136 through AA165 were deleted and replaced with the Ad43 HVR1 amino acids from AA136 through 153. The HVR2 residues from Ad5 between and including AA190 through AA192 were deleted and replaced with the Ad43 HVR2 amino acids from AA178 through 184. The HVR3 residues from Ad5 between and including AA212 through AA218 were deleted and replaced with the Ad43 HVR3 amino acids from AA204 through 209. The HVR4 residues from Ad5 between and including AA252 through AA258 were deleted and replaced with the Ad43 HVR4 amino acids from AA244 through 251. The HVR5 residues from Ad5 between and including AA271 through AA279 were deleted and replaced with the Ad43 HVR5 amino acids from AA264 through 271. The HVR6 residues from Ad5 between and including AA305 through AA310 were deleted and replaced with the Ad43 HVR6 amino acids from AA279 through 302. The HVR7 residues from Ad5 between and including AA418 through AA428 were deleted and replaced with the Ad43 HVR7 amino acids from AA410 through 420. The HVR8 residues from Ad5, AA435 through AA436, were deleted as the corresponding region in Ad43 does not contain these amino acids. The HVR9 residues from Ad5 between and including AA440 through AA451 were deleted and replaced with the Ad43 HVR9 amino acids from AA430 through 440.

The specific amino acid sequences of the modified regions of the hexon proteins used in this study are shown in Figures S1 and S2. For the Ad5H(2-2) chimera, the DE1 loop containing HVRs 1–6 of Ad5 (Ad5 hexon AA 132–315) and the FG1 loop containing HVRs 7–9 from Ad5 (Ad5 hexon AA 420–449) were replaced with the corresponding loops from Ad2 (Ad2 hexon AA 132–327 and Ad2 hexon AA 432–465). Similarly, the Ad5H(43–43) and Ad5H(34–34) chimeras contain deletions in the DE1 loop containing HVRs 1–6 of Ad5 (Ad5 hexon AA 132–315) and the FG1 loop containing HVRs 7–9 from Ad5 (Ad5 hexon AA 420–449). These loops were replaced with the corresponding loops from Ad43 and Ad34 respectively.

### Animals

Female 5 to 8 week old BALB/c AnNCr mice were purchased from either Harlan (Indianapolis, IN) or the National Cancer Institute (Frederick, MD). All rabbit studies reported herein were conducted under contract at Spring Valley (Woodbine, MD) in 1.5–2.5 kg (∼6 week old), female, New Zealand white rabbits purchased from Harlan.

### Neutralizing antibody assay

Our neutralizing antibody assay is based on the capacity of antibodies to neutralize adenovectors that express luciferase and is similar to that described by Sprangers et al., 2003 [63]. First, hexon-modified vectors that express luciferase were titrated to determine the dose response curve for luciferase activity. A549 cells (American Type Culture Collection (ATCC) Manassas, VA) were infected with these vectors and a control Ad5-based vector that expresses luciferase (Ad5L) at multiplicities of infection (MOI) between 10 and 4000 pu/cell. Luciferase activity was monitored from cell lysates harvested 24 hours after infection. The luciferase curves generated by the hexon-modified vectors were similar to the control Ad5-based vector. Next we used the newly constructed luciferase-expressing chimeras at an MOI in the linear range of the assay, typically 500 pu/cell, to determine whether Ad5 NAb is capable of neutralizing these chimeric hexon-containing vectors.

High titer Ad5 NAb sera were generated in mice and rabbits. BALB/c mice or NZW rabbits were primed with 1×10^10^ pu of adenovector and then boosted with 1×10^10^ pu of the same vector 4 weeks after the prime. Three weeks after the boost, blood was collected by cardiac puncture, allowed to clot at room temperature, and the serum collected and frozen at −80°C for subsequent use in the Ad5 NAb assay. Ad5 NAb were generated using Ad5, and hexon-specific Ad5 Nab were generated using a chimeric Ad5 vector that contains an Ad35 fiber, Ad5.F(35). Rabbit Ad5 NAb sera were prepared at Spring Valley Laboratories Inc., Woodbine, MD.

Pooled serum from 30 immunized mice, six individual rabbits, or individual humans was heat inactivated at 56°C for 60 minutes, serially diluted and mixed with the luciferase expressing adenovectors. After a 30 minute incubation at room temperature, the virus/serum mixture was used to infect A549 cells seeded on 96 well plates. Luciferase activity from individual wells was measured using a 96 well luminometer and the highest dilution of serum that resulted in 90% inhibition of luciferase activity was determined.

We used a one tailed Paired Sample Wilcoxon Signed Rank test to compare NAb titers in rabbit and human serum samples obtained with the four reporter adenovectors; Ad5L, Ad5L.H(43 m-43), Ad5L. F(35) and Ad5L.H(43 m-43).F(35). *P* values of less than 0.05 were considered significant.

### Immunizations

BALB/c mice were immunized with *Pf*CSP expressing adenovirus vector in a 0.1 ml volume by IM immunization (bilateral injections into tibialis anterior muscles with a 29*G*1/2 needle). Immunizations to generate pre-existing Ad5 neutralizing antibodies occurred by IM injection of adenovector into the left gastrocnemius muscle (1×10^10^ pu) followed by a booster immunization with the same vector (1×10^10^ pu) given 4 weeks later. These doses were selected based on results of dose titration studies. Experiments include groups of mice immunized with adenovector without the gene of interest (AdNull) or containing an irrelevant gene (Ad.L) and naïve non-immunized negative control mice. NZW rabbits (N = 6/group) were immunized by two 0.5 ml injections into the right quadricep muscles with 1×10^10^ particles of adenovirus vector using a 26*G* needle.

### Immunological Assays

Cellular immune responses were assessed by *ex vivo* IFNγ ELISpot assays (N = 6 mice/group, assayed in pools) or intracellular cytokine staining assays (N = 6 mice/group, assayed individually) as described previously [55], using splenocytes harvested from immunized or control mice as effector cells. Targets were MHC-matched A20.2J cells pulsed with synthetic peptides.

#### ELISpot

Splenocytes (group pools, tested in quadruplicate) were stimulated with peptide-loaded A20 cells for 36 hours. Target cells pulsed with an irrelevant peptide and unpulsed target cells served as negative controls. The number of IFNγ secreting cells, visualized as spots, was determined using an ELISpot Reader (AID, Autoimmun Diagnostika). Data are presented as the mean spot forming cells (SFC) ± standard deviation.

#### ICS

1×10^6^ splenocytes/mouse were stained for viability, CD3, CD4, CD8, IL-2, IFNγ, and TNFα. Splenocytes + peptide-loaded A20 cells were incubated for 2 hours, golgi-inhibited, incubated another 6 hr and then stained and fixed. All available cells were acquired (LSRII, BD Biosciences), then analyzed with FlowJo (Mac OSX version 8.8.6). Data are presented as mean percent cytokine-producers of total CD3+CD8+ T cells. Individual mouse data tested in singleton are represented as a single dot overlapping the mean. Statistical analysis of ICS data was performed using one way analysis of variance (ANOVA) followed by Bonferroni's means comparison test. *P* values of less than 0.05 were considered significant.

Multifunctional T cell analyses were graphed in SPICE, version 5.

Analysis and presentation of distributions was performed using SPICE version 5.2, downloaded from http://exon.niaid.nih.gov [64]. Comparison of distributions was performed using a Student's T test and a partial permutation test as described [64].

#### ELISA

Antibody responses were assayed by ELISA as described previously [55] against either *Py*CSP recombinant protein capture antigen or *Py*CSP repeat peptide(QGPGAP) using sera, assayed individually, from immunized or control mice. Endpoint titration *Py*CSP-specific antibody titers (OD 0.5) are shown, based on two-fold dilution curves of mouse serum (triplicate wells).

### Ethics Statement

The animal experiments reported herein as detailed in GenVec's Master Experimental Protocol (MP-BRU-8036-4-23-2007) were reviewed and approved by the GenVec Institutional Animal Care and Use Committee (IACUC). GenVec's IACUC ensures that all *in vivo* experimental protocols undergo a thorough review process to assure that all *in vivo* experiments conducted comply with all Animal Welfare Regulations and in accordance with the principles set forth in the “Guide for the Care and Use of Laboratory Animals”. GenVec's Animal Welfare Assurance number is A4398-01.

## Supporting Information

Figure S1 **Amino acid sequences of the vectors shown in** **Figure 1** **.** The amino acid sequences of the DE1 and FG1 loops, between and including position 126 through 461 of the Ad5 and specific chimeric hexons are indicated. Red highlighted amino acids indicate hypervariable regions of the Ad5 hexon [48]. Strike-through lines indicate amino acids that are not resolved in the Ad5 crystal structure. Underlined residues indicate amino acids that are different from Ad5 in the chimeras.
(TIF)

Figure S2 **Amino acid sequences of the vectors shown in** **Figure 2** **.** The amino acid sequences of the DE1 and FG1 loops, between and including positions 126 through 461 of the Ad5 and specific chimeric hexons are indicated. Yellow highlighted regions indicate Ad5 amino acid residues that are replaced with amino acids from Ad43 in the H(43 m-43) chimeric hexon. Underlined residues are amino acids that are different from Ad5.
(TIF)

Figure S3 **Capsid protein composition of wild-type Ad5, Ad5 vector and hexon-modified vectors by silver staining.** 2×10^9^ particles of wild-type Ad5, lane 2; Ad*Py*CSP, lane 3; Ad*Py*CSP.H(5–43), lane 4; Ad*Py*CSP.H(43 m-5), lane 5; Ad*Py*CSP.H(43 m-43), lane 6; were electrophoresed on a 4–12% SDS-polyacrylamide gel and proteins were stained with silver. Molecular weight markers were run in the first lane and their weights are indicated. The specific adenovirus proteins that correspond to the prevalent bands are indicated.
(TIF)

Figure S4 **Neutralization curves for Ad5L and Ad5L.H(43 m-43).** Sera obtained from mice immunized with an Ad5 vector, Ad5 (A) or an Ad5 vector with an Ad35 fiber replacement, Ad5.F35 (B) were diluted 1∶48 and then 3-fold serial dilutions were tested for neutralizing activity toward an unmodified Ad5L vector and the Ad5L.H(43 m-43) hexon-modified vector.
(EPS)

Figure S5 **Hexon-modified vector efficiently boosts Ad5 vector primed T cell and antibody responses.** BALB/c mice were first primed with 1×10^6^ pu (A) or 1×10^7^ pu (B) of Ad5*Py*CSP and boosted with the same dose of Ad5*Py*CSP.H(43 m-43) four weeks later. Controls included mice that were immunized with a single dose of Ad5*Py*CSP at day 0 or a single dose of Ad5*Py*CSP.H(43 m-43) administered at day 28. CD8^+^ IFNγ^+^ T cell responses were assessed two weeks after boost by ICS. (C) *Py*CSP specific antibody responses were analyzed by ELISA from sera collected 2 weeks after boost.
(TIF)

Table S1 **Physical vector particle: Active particle ratios (Pu/ffu) for adenovectors used in this study are shown.**
(DOCX)
