# Correction: Modification of Ad5 Hexon Hypervariable Regions Circumvents Pre-Existing Ad5 Neutralizing Antibodies and Induces Protective Immune Responses

**DOI:** 10.1371/annotation/c110beed-3cac-48db-9039-ba4498d5db50

**Published:** 2012-05-04

**Authors:** Joseph T. Bruder, Elena Semenova, Ping Chen, Keith Limbach, Noelle B. Patterson, Maureen E. Stefaniak, Svetlana Konovalova, Charlie Thomas, Melissa Hamilton, C. Richter King, Thomas L. Richie, Denise L. Doolan

Due to an error in production, an incorrect competing interests statement was published. The correct competing interests statement is:

The authors have read the journal's policy and have the following conflicts: Joseph T. Bruder has ownership of stocks and options and paid employment at GenVec, Inc. Elena Semenova has stock options and paid employment at GenVec, Inc. Ping Chen has ownership of stocks and options and paid employment at GenVec, Inc. Svetlana Konovalova has ownership of stocks options and paid employment at GenVec, Inc. Melissa Hamilton has ownership of GenVec, Inc. stock. The following authors have no competing interests: Keith Limbach, Noelle B. Patterson, Maureen E. Stefaniak, Thomas L. Richies and Denise L. Doolan. This does not alter the authors' adherence to all the PLoS ONE policies on sharing data and materials. 

