# Supplementary figures and images for: Modification of Ad5 Hexon Hypervariable Regions Circumvents Pre-Existing Ad5 Neutralizing Antibodies and Induces Protective Immune Responses
Source: PLoS One. 2012 Apr 5;7(4):e33920. doi: 10.1371/journal.pone.0033920 (PMC3320611; doi:10.1371/journal.pone.0033920)

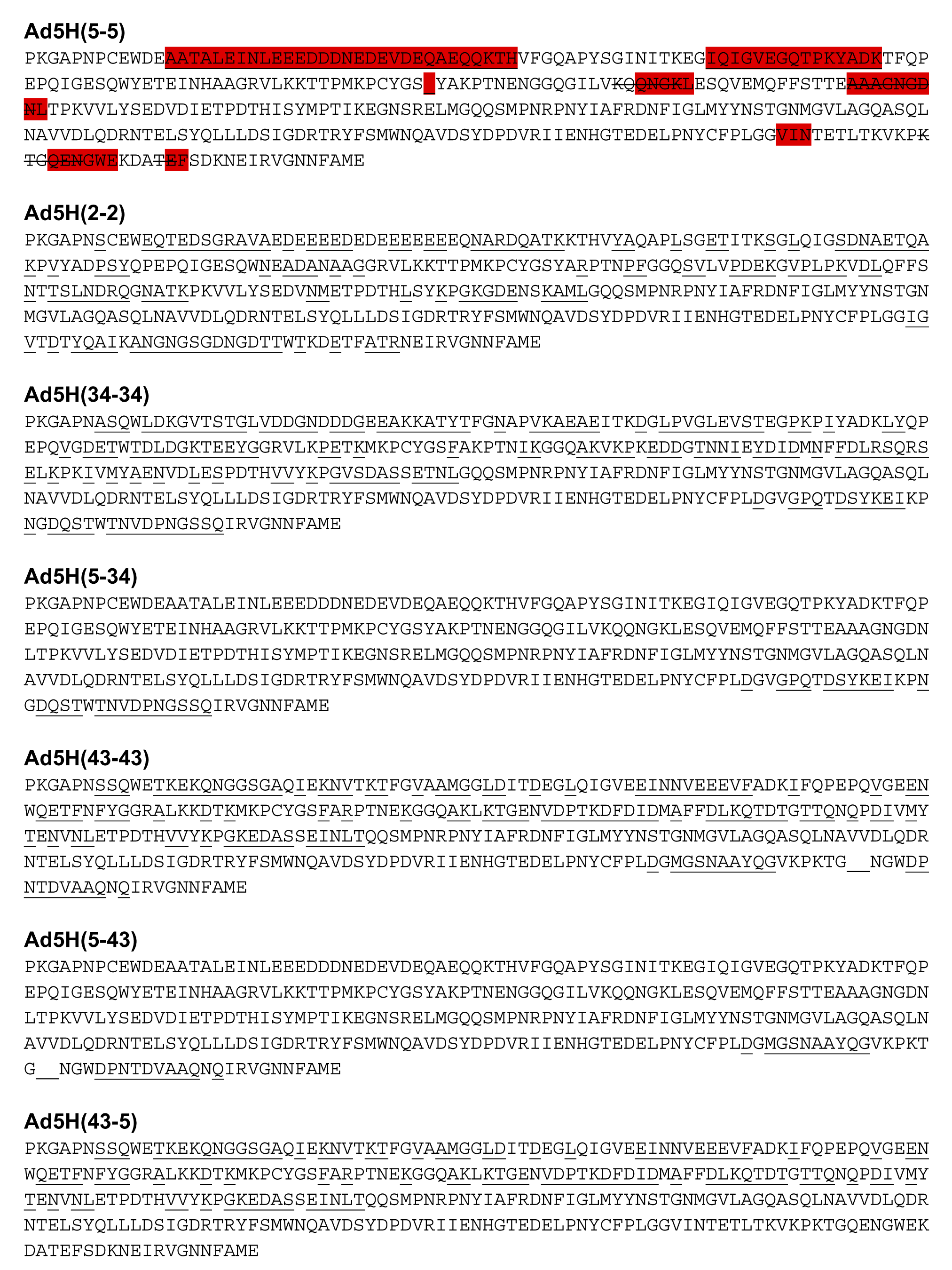

Supplement: Figure S1 — Amino acid sequences of the vectors shown in Figure 1 . The amino acid sequences of the DE1 and FG1 loops, between and including position 126 through 461 of the Ad5 and specific chimeric hexons are indicated. Red highlighted amino acids indicate hypervariable regions of the Ad5 hexon [48]. Strike-through lines indicate amino acids that are not resolved in the Ad5 crystal structure. Underlined residues indicate amino acids that are different from Ad5 in the chimeras. (TIF) [file pone.0033920.s001.tif]

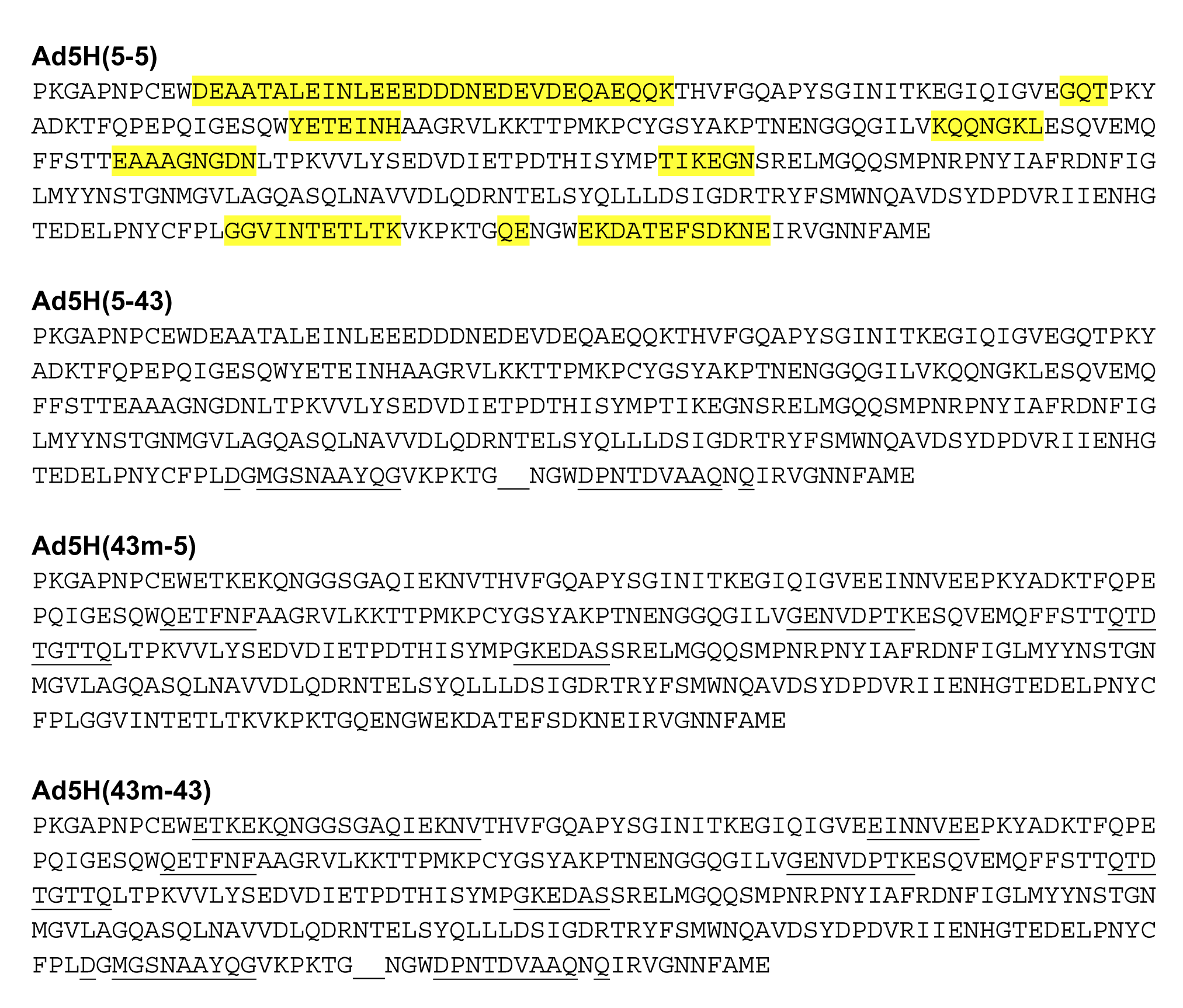

Supplement: Figure S2 — Amino acid sequences of the vectors shown in Figure 2 . The amino acid sequences of the DE1 and FG1 loops, between and including positions 126 through 461 of the Ad5 and specific chimeric hexons are indicated. Yellow highlighted regions indicate Ad5 amino acid residues that are replaced with amino acids from Ad43 in the H(43 m-43) chimeric hexon. Underlined residues are amino acids that are different from Ad5. (TIF) [file pone.0033920.s002.tif]

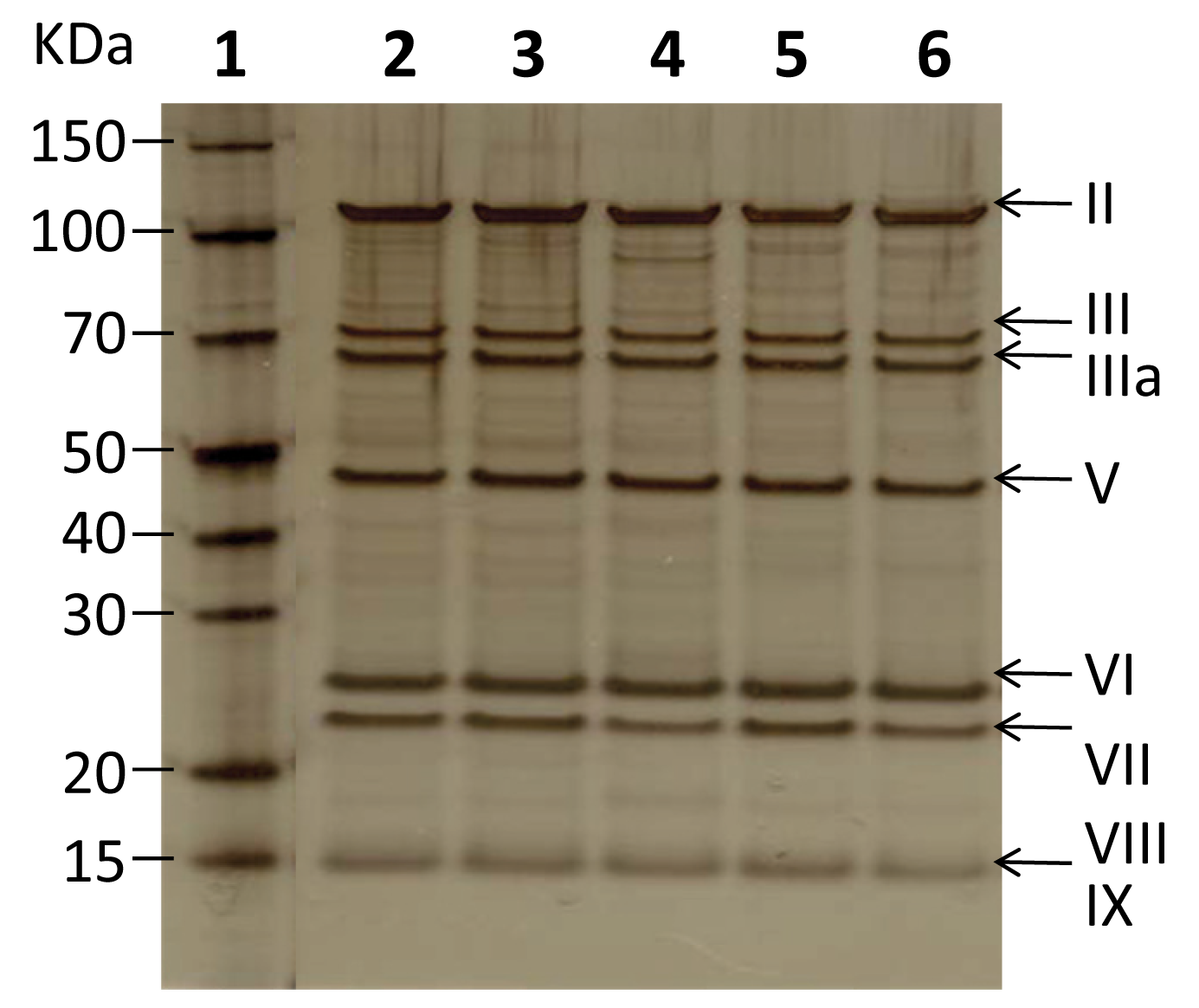

Supplement: Figure S3 — Capsid protein composition of wild-type Ad5, Ad5 vector and hexon-modified vectors by silver staining. 2×10^9^ particles of wild-type Ad5, lane 2; AdPyCSP, lane 3; AdPyCSP.H(5–43), lane 4; AdPyCSP.H(43 m-5), lane 5; AdPyCSP.H(43 m-43), lane 6; were electrophoresed on a 4–12% SDS-polyacrylamide gel and proteins were stained with silver. Molecular weight markers were run in the first lane and their weights are indicated. The specific adenovirus proteins that correspond to the prevalent bands are indicated. (TIF) [file pone.0033920.s003.tif]

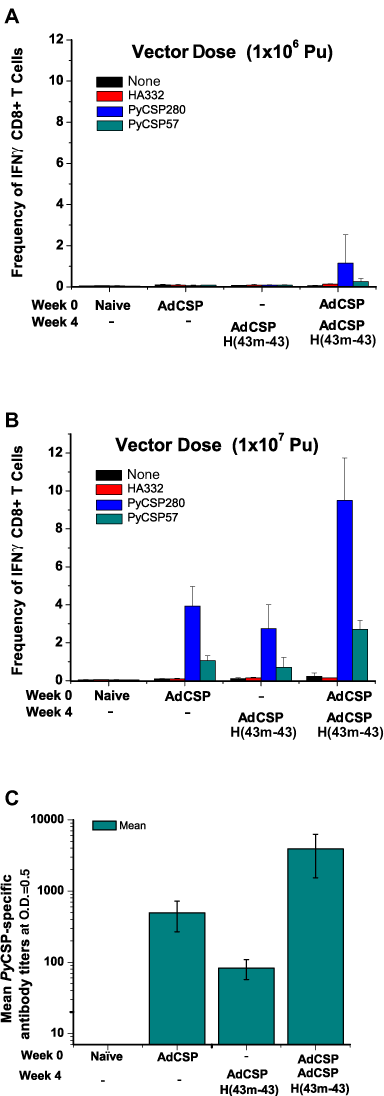

Supplement: Figure S5 — Hexon-modified vector efficiently boosts Ad5 vector primed T cell and antibody responses. BALB/c mice were first primed with 1×10^6^ pu (A) or 1×10^7^ pu (B) of Ad5PyCSP and boosted with the same dose of Ad5PyCSP.H(43 m-43) four weeks later. Controls included mice that were immunized with a single dose of Ad5PyCSP at day 0 or a single dose of Ad5PyCSP.H(43 m-43) administered at day 28. CD8+ IFNγ+ T cell responses were assessed two weeks after boost by ICS. (C) PyCSP specific antibody responses were analyzed by ELISA from sera collected 2 weeks after boost. (TIF) [file pone.0033920.s005.tif]
