# Supplementary material for: Modification of Ad5 Hexon Hypervariable Regions Circumvents Pre-Existing Ad5 Neutralizing Antibodies and Induces Protective Immune Responses
Source: PLoS One. 2012 Apr 5;7(4):e33920. doi: 10.1371/journal.pone.0033920 (PMC3320611; doi:10.1371/journal.pone.0033920)
Supplement: Table S1 — Physical vector particle: Active particle ratios (Pu/ffu) for adenovectors used in this study are shown. (DOCX) [file pone.0033920.s006.docx]

**Table S1**

**Particle: Active Particle Ratios of Vectors**

| Vector Name | Pu/ffu Ratio |
| --- | --- |
| AdPyCSP1 | 6 |
| Adt.PyCSP.H(2-2) | 12 |
| Adt.PyCSP.H(5-43) | 31 |
| Adt.PyCSP.H(5-34) | 34 |
| Adt.PyCSP.H(43m-43) | 18 |
| Adt.PyCSP.H(43m-5) | 11 |
|  |  |
| AdL | 6 |
| Adt.L H(2-2) | 44 |
| Adt.L H(5-34) | 26 |
| Adt.L H(5-43) | 14 |
| Adt.L H(43m-5) | 16 |
| Adt.L H(43m-43) | 9 |
| Adt.L.H(43m-43).F35 | 11 |
| AdL.F35 | 12 |
